# Sensitivity of Piezoelectric-Based Smart Interfaces to Structural Damage in Bolted Connections

**DOI:** 10.3390/s19173670

**Published:** 2019-08-23

**Authors:** Thanh-Canh Huynh, Duc-Duy Ho, Ngoc-Loi Dang, Jeong-Tae Kim

**Affiliations:** 1 Faculty of Civil Engineering, Duy Tan University, 03 Quang Trung, Hai Chau, Danang 550000, Vietnam; 2 Center for Construction, Mechanics and Materials, Institute of Research and Development, Duy Tan University, 03 Quang Trung, Hai Chau, Danang 550000, Vietnam; 3 Faculty of Civil Engineering, Ho Chi Minh City University of Technology, VNU-HCM, 268 Ly Thuong Kiet, District 10, Ho Chi Minh City 700000, Vietnam; 4 Ocean Engineering Department, Pukyong National University, 45 Yongso-ro, Daeyeon 3-dong, Namgu, Busan 48513, Korea

**Keywords:** lead zirconate titanate (PZT), piezoelectric interface, piezoelectric sensors, sensitivity, impedance method, damage detection, impedance responses, geometric effect, bolted connection, loosened bolt

## Abstract

This study presents a set of experimental and numerical investigations to study the sensitivity of the piezoelectric-based smart interface device to structural damage in a bolted connection. The study aims to identify the proper geometric sizes of smart interfaces for damage detection tasks. First, the fundamentals of the damage monitoring technique via lead zirconate titanate (PZT) interface is briefly described for a bolted connection. Second, a lab-scaled girder connection is selected as the test structure for the experimental investigation. PZT interface prototypes with varying geometric sizes are designed for the test connection. Under the bolt-loosening inflicted in the connection, the impedance responses of the PZT interfaces are analyzed to understand the effect of geometric parameters on the damage sensitivity of the impedance responses. Subsequently, the bolt-loosening detection capabilities of the PZT interfaces are comparatively evaluated for identifying the proper geometric sizes of the devices. Finally, a finite element model of the PZT interface-bolted connection system is established for the numerical investigation. The damage sensitivity of the numerical impedance responses is compared with the experimental results for the verification.

## 1. Introduction

Civil structures are important facilities that support the economic and social activities of a nation. During their operation, these structures are often exposed to severe environmental and loading conditions. Extreme events, such as earthquakes and tsunamis, could result in inevitable structural deficiencies, causing seriously negative damage to social properties and loss of human life. Structural health monitoring (SHM) systems have been shown to have an important role in the integrity assessment of in-service civil structures. Basically, an SHM system implements a process of damage detection and characterization. The damage-induced sensitive features of the monitored structure are recorded via the SHM system, and the health status of the structure is assessed by analyzing these features.

Critical bolted connections are concerned to be the ‘*hot spots*’ in steel structures. Bolted connections that are poorly maintained, in fact, are originators that could lead to serious failures of the load-bearing elements, or even the collapse of the entire structural system. Many studies have been carried out to develop efficient SHM tools for damage assessment of critical joints, including a vibration/ultrasonic-based method, a vision-based method, a guided wave-based method, and an impedance-based method [1,2,3,4,5,6,7,8]. Among recent SHM technologies to monitor critical joints, the impedance-based method has been emerging as a cost-effective and innovative tool, with many successful applications in laboratory and field scales [5,9,10,11,12,13,14]. Importantly, the impedance-based damage detection algorithm can be embedded into smart sensor nodes to perform real-time and wireless SHM, as well as damage detection tasks [15,16,17,18,19,20].

Typically, the impedance-based method uses a cheap, fast-response, non-intrusive, and linear piezoelectric (i.e., lead zirconate titanate (PZT)) patch to acquire the electromechanical (EM) impedance signatures from a target structure. Subsequently, damage-sensitive features, such as frequency shifts, amplitude changes, and statistical damage metrics, are extracted from the EM impedance signatures and used for damage detection. Traditionally, the PZT patch is permanently bonded to the host structure (directly bonded attachment) in the impedance-based SHM system. This often causes barriers in the desired modification of an existing sensor placement, and influences the repeatability of impedance signals [21,22]. More importantly, the direct attachment method often leads to weak resonant impedance responses that indeed influence the accuracy of the damage monitoring. As a consequence of this, selecting proper frequency bands in the weak impedance zone becomes challenging for damage detection tasks.

To deal with the above-mentioned issues, piezoelectric-based portable devices have been developed and recently gained significant attention from SHM academy. Annamdas et al. [21] developed a method to indirectly obtain the impedance responses by inserting an additional structural element at the interface between the PZT and the monitored structure. The authors’ idea was an originator to later developments of portable piezoelectric devices. Na et al. [23] proposed an idea for creating resonant frequencies of impedance signals by attaching the PZT patch on a metal disc. Wang et al. [24] designed a wearable, PZT-based ring for health monitoring of bolted joints in a pipeline system. Huynh et al. [25] developed the portable PZT interface device by embedding a PZT into a fixed-fixed beam structure. The major advantages of Huynh et al.’s technique include easy predetermination of a damage-sensitive frequency range using simple equations [26], as well as portability that allows the interface to be easily attached and detached from a target joint. The practicality of the PZT interface technique was evidenced via a health assessment of critical joints under conditions of constant and varying temperatures [27,28].

Owning to its advantages, the PZT interface technique has been applied to pre-determine the damage-sensitive frequency bands for damage monitoring of bolted connections [29,30]. Although the PZT interface technique has shown promising results, the effect of the PZT interface’s geometric parameters on the measured impedance signatures has not been intensively understood. Basically, the geometric sizes of the PZT interface are important parameters, which directly influence the sensitivity of impedance responses to structural damage. To maximize the damage detection performance, the geometric parameters of the PZT interface should be designed properly.

This study presents a set of experimental and numerical investigations to study the influence of a PZT interface’s geometric parameters on the damage sensitivity of the EM impedance. First, the PZT interface-based impedance monitoring technique for bolted joints is briefly described. Second, a lab-scaled girder connection is selected as the host structure of the PZT interface for the experimental investigation. A few PZT interface prototypes, which are different in geometric size and boundary conditions, are designed for the tested girder connection. In the damage cases, the impedance responses of the different PZT interfaces are recorded. The damage sensitivity of the impedance responses is analyzed to determine the suitable sizes of the PZT sensor and the interface for damage detection. Finally, the impedance response of the PZT interface-bolted connection system is numerically simulated, using the finite element method to confirm the experimental observations. The damage sensitivity of the numerical impedance responses is analyzed for the different PZT interfaces and compared with the experimental results.

## 2. Piezoelectric Interface-Based Damage Monitoring Technique for Bolted Joints

**Figure 1:**
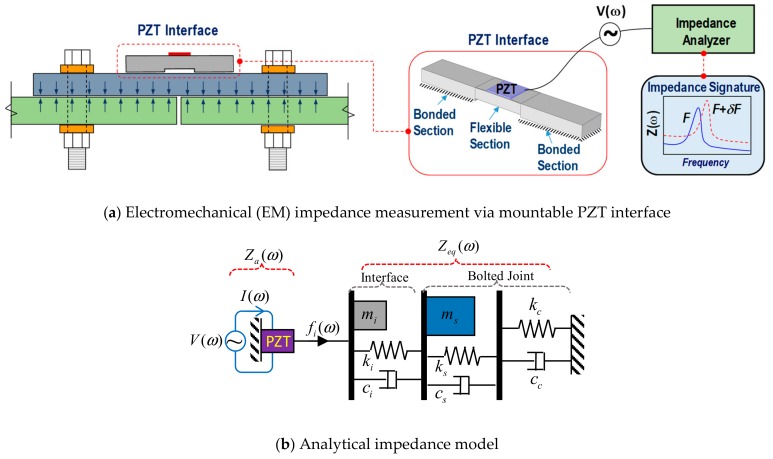
Illustration of the piezoelectric-based, smart interface technique for damage monitoring in a bolted joint.

The piezoelectric-based, smart interface technique has been proposed to pre-determine the sensitive frequency bands in the damage monitoring of a bolted connection [29]. The interface is a substrate structure that is embedded with a PZT sensor, as shown in Figure 1a. The PZT interface can be easily attached and detached from the bolted connection via two outside bonded sections. The middle of the interface is a flexible section that allows easy elongation of the PZT under an applied voltage. The flexible section is designed to easily create resonant impedance responses. Figure 1a schematizes the impedance measurement of the bolted connection via the PZT interface. The PZT is excited by a high-frequency harmonic voltage via an impedance analyzer. An output current, due to the piezoelectric effect, is then measured for computing the EM impedance in a frequency domain.

As the bolts are loosened, the contact parameters (including contact stiffness and contact damping) at the interface between the connecting members are altered [31,32]. This alternation will modify the coupling behaviors between the PZT interface and the bolted joint under PZT excitation, leading to the shifts in measured impedance signatures. By comparing the pre- and post-damage impedance signatures, the bolt looseness occurring in the joint can be detected.

Figure 1b shows an analytical impedance model of the PZT-interface bolted joint system that can be used to explain how the PZT interface method is feasible for damage detection. As shown in the figure, one of the degrees of freedom (DOF) refers to the interface (*m_i_*, *c_i_*, *k_i_*), and the other represents the host structure (*m_s_*, *c_s_*, *k_s_*). The effect of bolt torque is simulated by the contact damping and contact stiffness in the connection (*c_c_*, *k_c_*). Under the voltage-induced harmonic elongation of the PZT, a harmonic excitation force *f_i_*(*ω*) is generated and drives the interface-bolted connection system, making the interface couple with the bolted joint. The structural impedance of the entire system *Z_eq_* is computed as [29]

(1) $$Z_{eq}=\frac{\left(-\omega^{2}m_{i}+j\omega c_{i}+k_{i}\right)\left(-\omega^{2}m_{s}+j\omega (c_{i}+\frac{c_{s}c_{c}}{c_{s}+c_{c}})+(k_{i}+\frac{k_{s}k_{c}}{k_{s}+k_{c}})\right)-{\left(j\omega c_{i}+k_{i}\right)}^{2}}{j\omega \left(-\omega^{2}m_{s}+j\omega (c_{i}+\frac{c_{s}c_{c}}{c_{s}+c_{c}})+(k_{i}+\frac{k_{s}k_{c}}{k_{s}+k_{c}})\right)}$$

where *j* signifies the imaginary unit and *ω* is the angular frequency of the voltage excitation.

The total EM impedance of the PZT interface as coupled with the bolted connection is computed as [26,33]

(2) $$Z(\omega )={\left\{j\omega \frac{w_{a}l_{a}}{t_{a}}\left[\varepsilon_{33}^{T}(1-j\delta )-d_{31}^{2}(1+j\eta )Y_{11}^{E}+\frac{Z_{a}(\omega )}{Z_{a}(\omega )+Z_{eq}(\omega )}d_{31}^{2}(1+j\eta )Y_{11}^{E}\frac{\tan(kl_{a})}{kl_{a}}\right]\right\}}^{-1}$$

in which *w_a_*, *l_a_*, and *t_a_* signify the length, width, and thickness of the PZT, respectively; $Y_{11}^{E}$ is the Young’s modulus of the PZT material at constant electric fieldl $\varepsilon_{33}^{T}$ is electric permittivity of the PZT material at constant stress; $d_{31}$ is the piezoelectric strain coefficient; *η* and *δ* denote the mechanical loss factor and the dielectric loss factor of the PZT material, respectively; and *k, ω*, and *Z_a_* are wave number, angular frequency, and mechanical impedance of the PZT material, respectively. Equations (1) and (2) theoretically demonstrate that the measured impedance signatures will contain the structural information of the whole system, including the structural properties and contact parameters of the connection. As the PZT interface’s structural parameters are assumed to be unchanged, alternation in the measured impedance signatures should be an indicator for the structural damage existing in the bolted connection. 

For damage detection, a well-known statistical damage index, the so-called root-mean-square deviation (RMSD), is often used to quantify the damage-induced variations in impedance responses. The RMSD index is defined as follows [34]:

(3) $$RMSD(Z,Z^{*})=\sqrt{\sum_{i=1}^{N}[Z^{*}(\omega_{i})-Z(\omega_{i}){]}^{2}/\sum_{i=1}^{N}[Z(\omega_{i}){]}^{2}}$$

where *Z*(*ωi*) and Z*(*ω_i_*) are the pre- and post-damage EM impedances at the *i*^th^ angular frequency, respectively, and *N* denotes the number of swept frequency points. When the structure is not damaged, the impedance signatures *Z*(*ωi*) and Z*(*ω_i_*) are almost similar, leading to an ignorable value for the RMSD. In contrast, the RMSD value will become significant as the structure is damaged. 

## 3. Experimental Investigation on Damage Sensitivity of Piezoelectric-based Smart Interface

### 3.1. Experiments on Bolted Connection

#### 3.1.1. Test Setup

**Figure 2:**
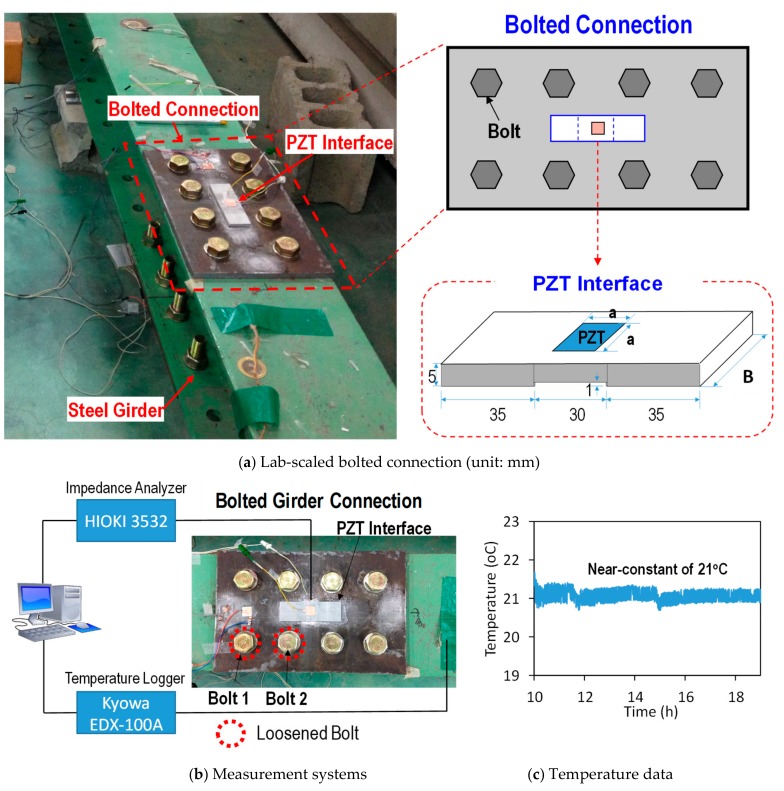
Test setup of the bolted joint for experimental investigation.

For the experimental analysis of the PZT interface’s geometric parameters, the bolted connection of the lab-scaled steel girder structure was selected as the target structure, as sketched in Figure 2a. We considered the bolted joint as a test structure for impedance monitoring, because of the ease of introducing gradual damages into the joint and the repeatability of the experiment. The two steel beams having H-shaped sections (H = 200 mm × 180 mm × 8 mm × 10 mm) are joined by splice plates (310 mm × 200 mm × 10 mm) and eight bolts (20 mm diameter, Korean Standard) at the top and bottom flanges. The PZT interface prototype was surface-mounted to the middle of the top splice plate, as shown in Figure 2a. By considering the size of the target joint, a PZT interface prototype was designed with the length of 100 mm, as well as a thickness of 5 mm for the bonded sections and 4 mm for the flexible section, as sketched in Figure 2a. The square PZT (PZT-5A type) has a size of *a* × *a* mm, and the interface has a width of *B* mm; the flexible section’s length is 30 mm, and the bonded section’s length is 35 mm.

The test setup of the connection is shown in Figure 2b. To acquire the impedance signatures, the PZT patch of the interface was excited by a 1 V harmonic excitation voltage using the HIOKI 3532 analyzer. The data logger Kyowa EDX-100A was used to acquire the connection’s temperature via the K-thermocouple wire. The PZT interface was mounted to the bolted joint via an instant adhesive layer (Loctite 401). The room temperature was controlled at around 21 °C during the experiment by air conditioners, as shown in Figure 2c. The temperature influence on the impedance responses of the PZT interface was quantified in the previous study [35]. The temperature variation will modify not only the resonant frequency, but also the peak magnitude of the impedance responses. Therefore, the temperature was kept near-constant to accurately quantify the effect of geometric sizes of the PZT interface on the impedance responses.

#### 3.1.2. Selection of PZT Interfaces

Among various geometric parameters, the size of PZT (i.e., *a*) and the width of the interface (i.e., *B*) were selected to vary. This is because the width of the interface deeply determines the local dynamic behaviors of the interface, while the size of the PZT is heavily associated with the piezoelectric driving force on the interface (the excitation capability) [36]. As listed in Table 1, four different interfaces (Interfaces 1–4) and four different PZT (PZTs 1–4) were selected to analyze the effect of the geometric parameters on the damage sensitivity of the impedance signatures. 

**Figure 3:**
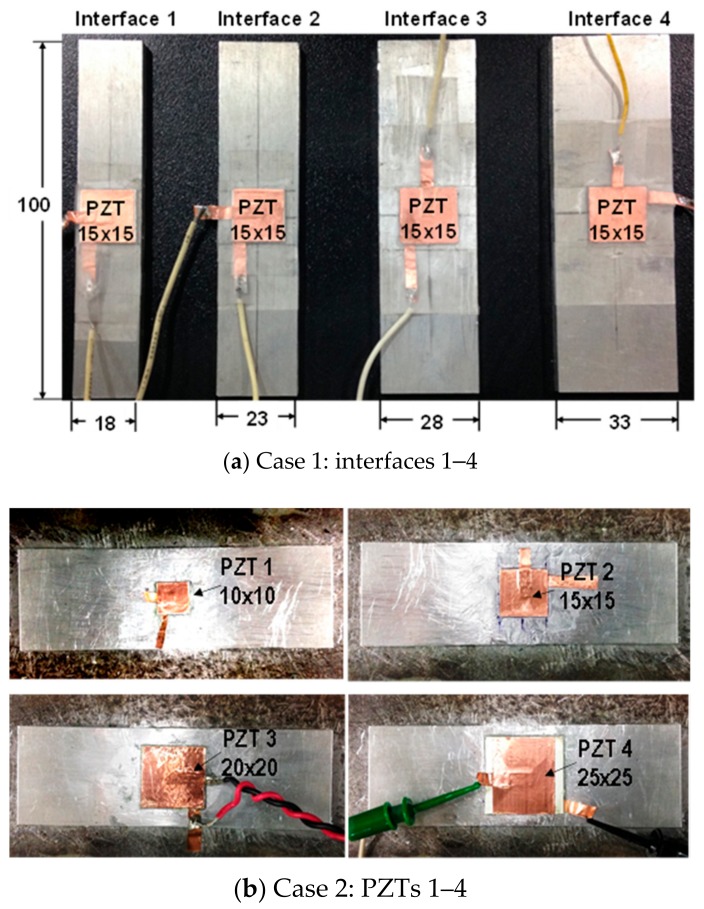
Real views of selected PZT interfaces (unit: mm).

In Case 1, to examine the effect of the interface’s size, the width (i.e., *B*) of the interface was varied from 18 mm to 33 mm, with an interval of 5 mm (interfaces 1–4), while other geometric parameters of the PZT interface prototype remained unchanged, as shown in Figure 3a. Interfaces 1–4 were embedded with the same PZT patches of 15 mm × 15 mm. The four interfaces have the following dimensions: 18 mm × 100 mm (Interface 1), 23 mm × 100 mm (Interface 2), 28 mm × 100 mm (Interface 3), and 33 mm × 100 mm (Interface 4). It should be noted that interface 1 is near the beam-like structure, while Interface 4 is close to the plate-like structure. Additionally, the four interfaces have different boundary conditions, owing to different bonding areas.

In Case 2, to examine the effect of the PZT’s size, the edge (i.e., *a*) of the square PZT was changed from 10 mm to 25 mm, with an interval of 5 mm (PZTs 1–4), while the other dimensional parameters of the PZT interface prototype were unchanged, as shown in Figure 3b. The four PZT sensors have the same thickness of 0.51 mm, but different dimensions: 10 mm × 10 mm (PZT 1), 15 mm × 15 mm (PZT 2), 20 mm × 20 mm (PZT 3), and 25 mm × 25 mm (PZT 4). As the size of the PZT sensor increases, the piezoelectric driving force on the interface device also increases, according to the reference [36].

It is worth noting that the PZT bonding condition will affect to the stress and strain transfer from the PZT to the interface via shear lag mechanism, and could accordingly influence the damage sensitivity of the PZT interface. In the previous parametric study, however, Islam and Huang [37] showed that if the host structure experiences flexural deformations (the flexural motions of the interface in this study), the effect of the bonding layer on the impedance resonances is slight and can be neglected. In another study, Bhalla and Moharana [38] also concluded that the mass of the bonding layer plays a negligible role and can be neglected. From these literature reviews, it is found that the PZT bonding condition may have slight effects in our test cases. The thickness of the bonding layer was not measured, due to the limited lab test conditions. Nonetheless, during the process of preparing individual interfaces, we have tried to maintain the uniform bonding condition of the PZT by considering the following issues: (1) the process of mounting PZTs were done by the same person with the same mounting method; (2) after attaching the PZTs to their desired positions, the same pressure was applied to the top surface of the PZT by adding a block mass; (3) after experiments, the PZTs were taken off, and their bonding conditions were visually checked.

#### 3.1.3. Testing Procedure

In Case 1, Interfaces 1–4 were sequentially mounted on the connection, and the impedance responses were obtained from the interfaces. In Case 2, while Interface 4 was still mounted on the connection, PZTs 1–4 were sequentially installed on the interface, and the impedance measurements were conducted. 

To examine the damage sensitivity of the impedance responses of the PZT interface, the bolt-loosening events of bolt 1 and bolt 2 (see Figure 2b) were simulated for both Case 1 and Case 2. The tested splice connection had eight bolts, which were symmetrically distributed over the splice plate (two bolts for each quarter of the splice plate). Based on the assumption that the connection was completely symmetric, only two bolts were therefore considered to be loosened in the test.

A torque wrench was used to fasten the bolts and to control the bolt torques. In this study, the authors considered the situation that the bolt torque was gradually decreased until 100% torque loss. The simulation was made for the realistic situation when a bolt is loosened under the effect of mechanical shocks and vibration [39]. The loss of torque will cause a significant reduction in the connection’s stiffness, leading to the instability of the whole structure. In our experiment, the connection had 16 bolts in total (8 for each splice plate). The loss of one or two bolts would not significantly affect to the stability of the whole beam.

At first, all bolts were fastened to 160 Nm for a healthy state. Then bolt 1 was loosened to 110 Nm (31% torque change), 60 Nm (62% torque change), and 0 Nm (100% torque change), sequentially. Next, bolt 1 was refastened to 160 Nm. Then, Bolt 2 was loosened to 110 Nm (31% torque change), 60 Nm (62% torque change), and 0 Nm (100% torque change), sequentially. The preload changes in the bolted connection are detailed in Table 2. After the bolt torque had reached the desired level, five repeated measurements of the impedance signatures were conducted simultaneously. 

### 3.2. Effect of Interface’s Geometric Parameters on Damage Sensitivity of Impedance Responses

#### 3.2.1. Impedance Signatures versus Interface Width

**Figure 4:**
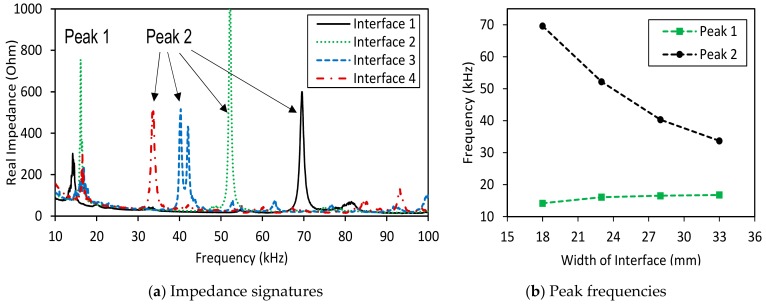
Impedance signatures under a healthy state of bolted girder connection: Interfaces 1–4.

The impedance signatures in 10–100 kHz (901 swept points) obtained from four PZT interfaces under a healthy state are shown in Figure 4a. Several resonant impedance peaks can be seen within the frequency range below 100 kHz. Two dominant impedance peaks (i.e., Peak 1 and Peak 2) are observed for each interface. As shown in Figure 4a, the first peaks (Peak 1) of the four interfaces were very close in their frequencies; meanwhile, the second peaks (Peak 2) were quite different from one another. It can be observed that the behaviour of Peak 1 was relatively unclear compared with that of Peak 2. This is due to the heavy modal damping of the excited vibration mode of Peak 1. It is known that the heavier damping of the excited vibration mode will cause the lower and less clear peak in the impedance signature.

The changes in frequencies of the two impedance peaks (Peak 1 and Peak 2) were plotted according to the increment of the interface’s width, as shown in Figure 4b. When the width of the interface was increased from 18 mm to 33 mm, the frequency of Peak 1 was slightly increased from 14.2 kHz to 17.2 kHz, while the second peak was significantly reduced from about 69.6 kHz to around 33.3 kHz. 

**Figure 5:**
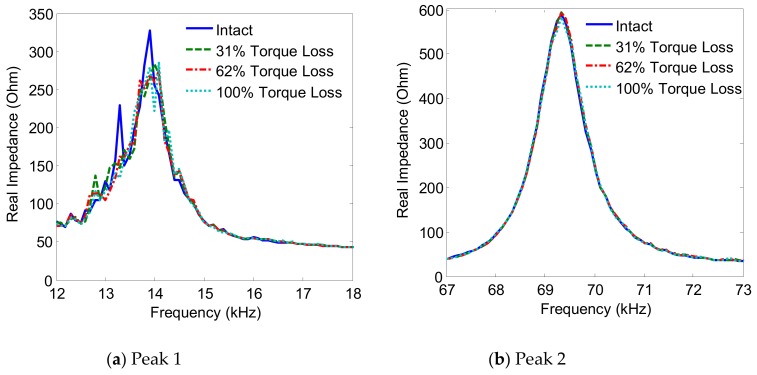
Impedance signatures under bolt 2 loosening events: Interface 1.

**Figure 6:**
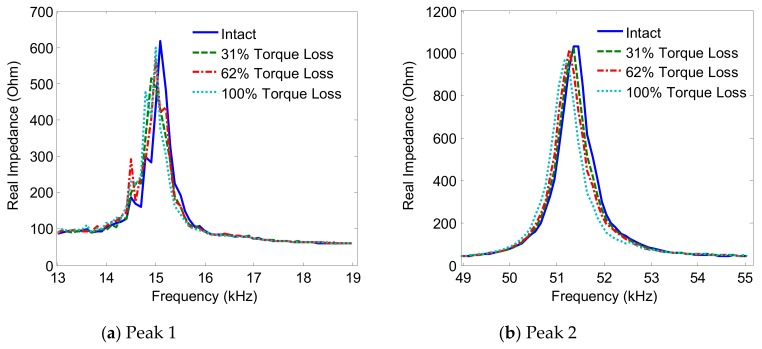
Impedance signatures under bolt 2 loosening events: Interface 2.

**Figure 7:**
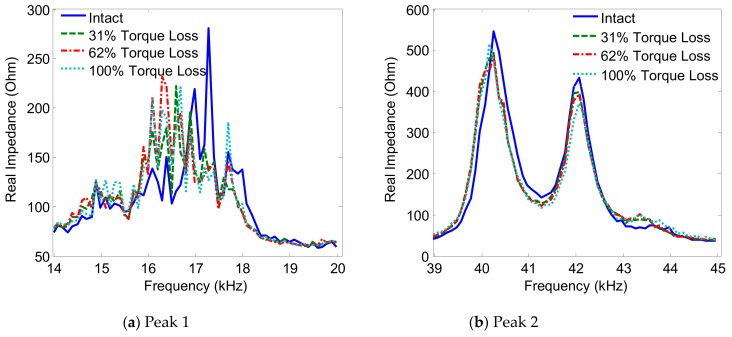
Impedance signatures under bolt 2 loosening events: Interface 3.

**Figure 8:**
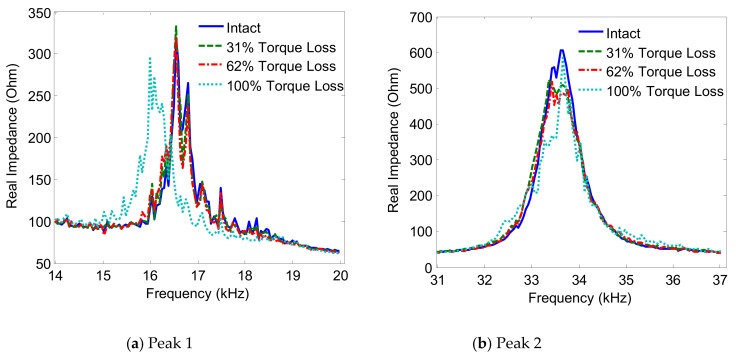
Impedance signatures under bolt 2 loosening events: Interface 4.

The variations of the two impedance peaks (Peak 1 and Peak 2) under the bolt 2 loosening events are shown in Figure 5, Figure 6, Figure 7 and Figure 8 for Interfaces 1–4, respectively. As observed from these figures, the impedance peaks tended to shift left as the torque is reduced, as the result of the reduction in the modal stiffness of the connection. Generally, Peak 1 showed more sensitivity to damage than Peak 2. The variation of the impedance signatures obtained from Interface 1 (beam-like interface) was relatively less significant than others. 

#### 3.2.2. Damage Sensitivity of Impedance Responses versus Interface Width

**Figure 9:**
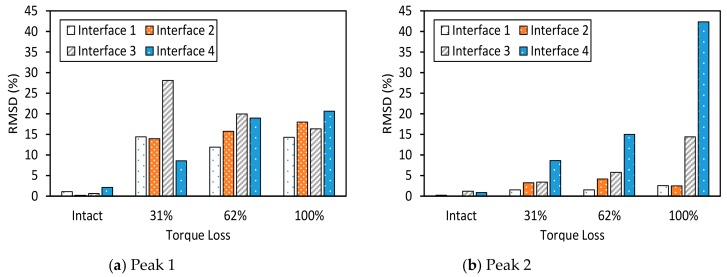
Root-mean-square deviation (RMSD) index of impedance signatures: bolt 1 loosening events.

**Figure 10:**
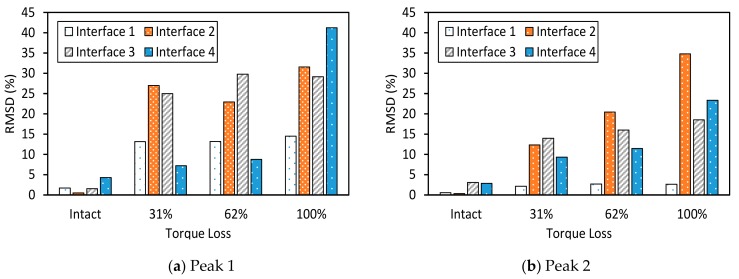
RMSD index of impedance signatures: bolt 2 loosening events.

The RMSD index of impedance signatures was computed under bolt 1 and bolt 2 loosening events, as shown in Figure 9 and Figure 10. Generally, the RMSD index increased according to the increment of the torque loss. For Peak 1, the RMSD index improved as the width of the interface increased, indicating that Peak 1 became more sensitive to the damage (see Figure 9a and Figure 10a). For Peak 2, the variation of the RMSD index induced by the bolt loosening was negligible in the case of Interface 1 (beam-like interface), but significant in other cases (plate-like interface), as revealed in Figure 9a and Figure 10b. This indicates that the damage sensitivity of the impedance signatures of Peak 2 was significantly improved when increasing the interface’s width. 

**Figure 11:**
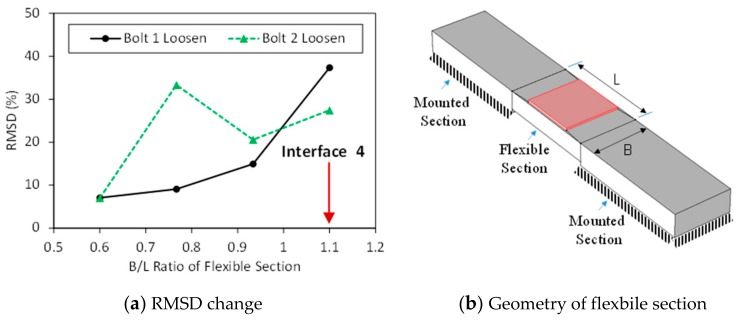
Sensitivity of impedance signatures versus interface size.

To estimate the suitable width of the interface, the RSMD index was computed by using the impedance signatures in 10–100 kHz. The RMSD index was plotted according to the variation of the width/length ratio (*B/L*) of the flexible section, as shown in Figure 11. It was found that Interface 4 with the *B/L* ratio of 1.1 provided the highest sensitivity of the impedance signatures to the bolt loosening. This means the plate-type flexible section of the interface could result in higher damage sensitivity than the beam-type flexible section.

### 3.3. Effect of PZT Size on the Damage Sensitivity of Impedance Responses

#### 3.3.1. Impendance Signatures versus Piezoelectric Size

**Figure 12:**
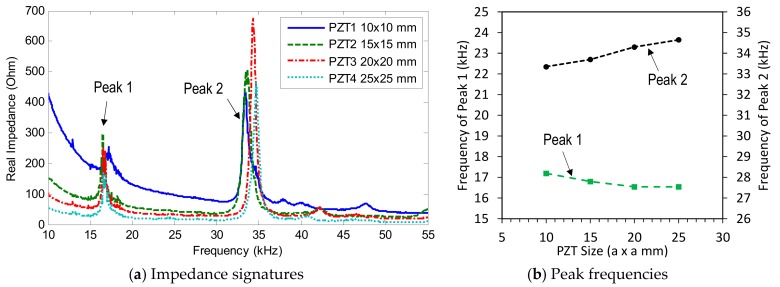
Impedance signatures under a healthy state of bolted girder connection: PZTs 1–4.

Figure 12a shows the intact impedance signatures in 10–55 kHz (901 swept points) measured from PZTs 1–4. As shown in the figure, the impedance pattern was shifted down as the PZT size increased, making sharper impedance peaks. Two impedance peaks (i.e., Peak 1 and Peak 2) can be seen within the examined frequency range. It can be observed that the four PZT sensors show impedance peaks with very close frequencies. As the PZT size was increased from 10 mm × 10 mm (PZT 1) to 25 mm × 25 mm (PZT 4), the first impedance frequency (Peak 1) slightly decreased, while the second one (Peak 2) slightly increased, as observed in Figure 12b. 

**Figure 13:**
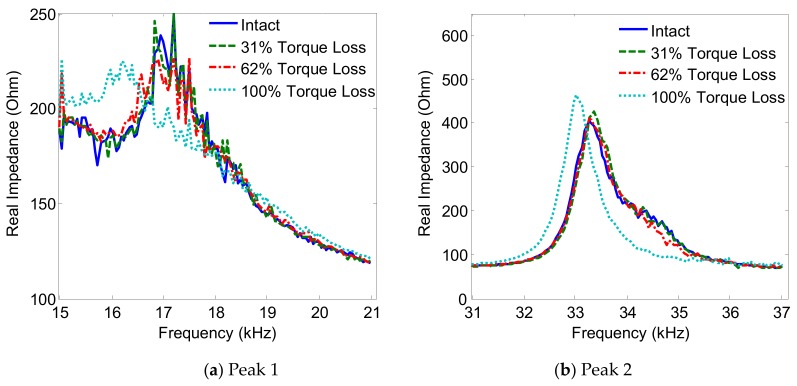
Impedance signatures under bolt 2 loosening events: PZT 1.

**Figure 14:**
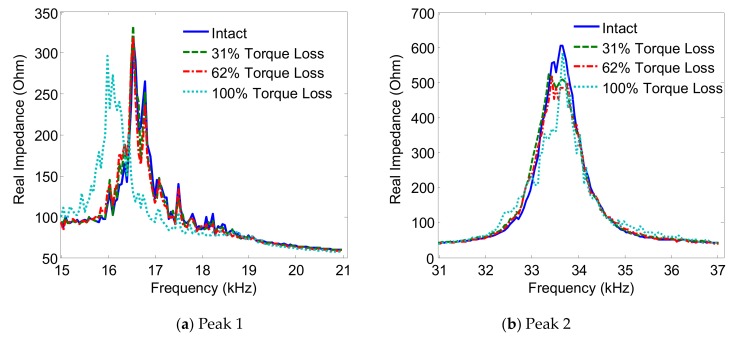
Impedance signatures under bolt 2 loosening events: PZT 2.

**Figure 15:**
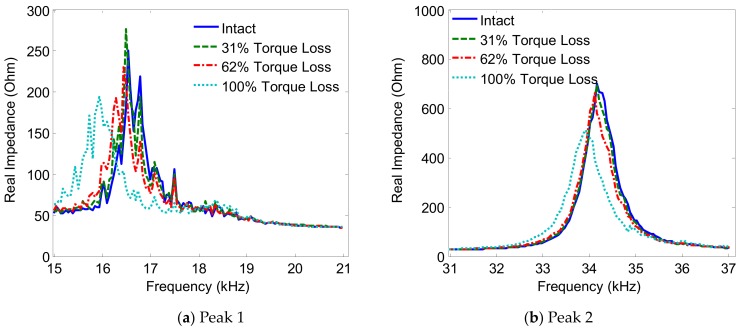
Impedance signatures under bolt 2 loosening events: PZT 3.

**Figure 16:**
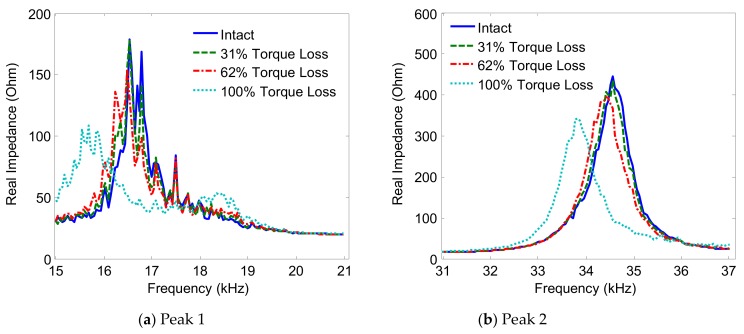
Impedance signatures under bolt 2 loosening events: PZT 4.

The variations of the impedance peaks (i.e., Peak 1 and Peak 2) under bolt 2 loosening events are shown in Figure 13, Figure 14, Figure 15 and Figure 16 for PZTs 1–4, respectively. As observed from these figures, the resonant impedance peaks tend to shift left as the torque is reduced, indicating the reduction of the modal stiffness of the connection. Generally, it was found that the variation of the impedance signatures obtained from the larger PZT sensors was more significant than that from the smaller sensors. As the PZT increased in size from 10 mm × 10 mm to 25 mm × 25 mm, the driving force on the interface was also increased 6.25 times, computed according to the reference [36]. This increased driving force of the PZT made stronger excitations to the target structure via the interface.

#### 3.3.2. Damage Sensitivity of Impedance Signatures versus PZT Size

**Figure 17:**
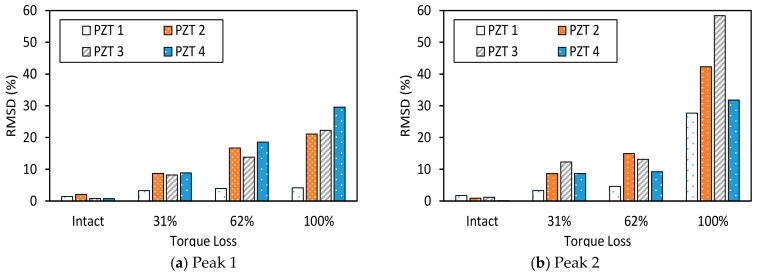
RMSD index of impedance signatures under bolt 1 loosening events: PZTs 1–4.

**Figure 18:**
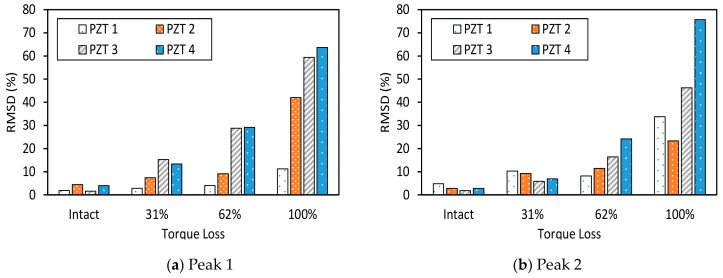
RMSD index of impedance signatures under bolt 2 loosening events: PZTs 1–4.

The RMSD index of the EM impedance signatures was computed under bolt 1 and bolt 2 loosening events for PZTs 1–4. The RMSD values of the four PZT sensors were compared, as shown in Figure 17 and Figure 18. For PZTs 1–4, the RMSD indices showed rapidly-increasing magnitude, according to the severity of the torque loss. Under the bolt 1 and bolt 2 loosening events, the RMSD indices of Peak 1 and Peak 2 increased when the PZT size was altered from 15 mm × 15 mm to 25 mm × 25 mm. This indicates that the sensitivity of the impedance signatures was improved by increasing the PZT’s size. It was found that PZT 1 was unable to detect bolt torque loss that was less than 62%. This is because of the weak piezoelectric driving force, due to the small size of the PZT. 

**Figure 19:**
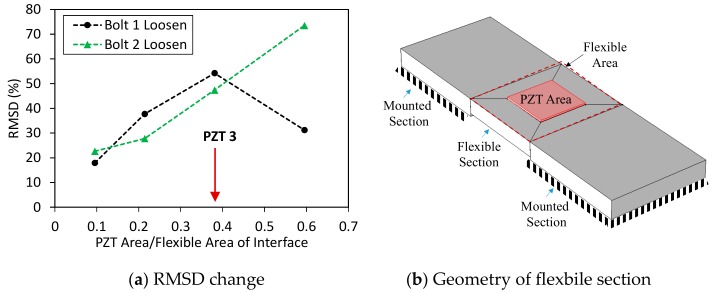
Sensitivity of impedance signatures versus PZT size.

To estimate the suitable size of the PZT, the RSMD index was computed by using the impedance signatures in the whole frequency range of 10–100 kHz. The RMSD index was plotted according to the ratio of surface areas between the PZT and the flexible section, as shown in Figure 19. It was found that PZT 3, which covers about 40% of the flexible section’s surface, provided the highest sensitivity of the impedance signatures to the bolt-loosening events.

## 4. Numerical Investigation on the Damage Sensitivity of Piezoelectric-Based Smart Interfaces

From the previous experimental investigation, it was shown that when increasing the width of the interface, as well as the PZT size, the damage sensitivity of impedance responses was improved. In this section, a finite element (FE) model of the test model was established to numerically analyze the influence of the PZT interface’s geometry, and to verify the experimental results.

### 4.1. Numerical Modeling of Bolted Connection

#### 4.1.1. Modeling of PZT Interface-Bolted Connection Interaction

**Figure 20:**
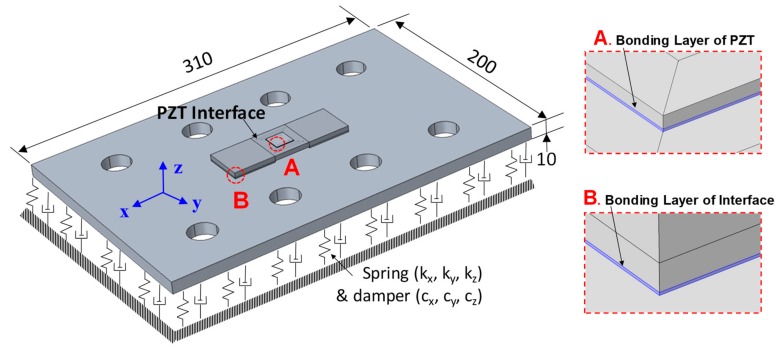
Finite element (FE) modeling of a bolted girder connection embedded with PZT interface (unit: mm).

The simulation of piezoelectric effects of the PZT interface requires electrical–mechanical coupled physics, which act simultaneously during the PZT excitation. The FE modeling of the PZT interface-bolted connection was carried out using COMSOL, which is commonly accepted by many researchers due to its electrical–mechanical simulation capabilities for modeling piezoelectric effects [40,41,42,43]. As discussed previously, the bolt preload of the bolted girder connection can be simulated by a system of contact spring and dashpot [31,32]; the FE model is therefore simplified by a splice plate on a three-dimensional spring (represented by *k_x_*, *k_y_*, and *k_z_*) and dashpot (represented by *c_x_*, *c_y_*, and *c_z_*) system, as shown in Figure 20a. The loss of the bolt preload (i.e., bolt-loosening) was simulated by the reduction of the contact stiffness. The splice plate and the PZT interface were modeled according to the actual sizes of the connection. The piezoelectric elements are used to model the piezoelectric effect of the PZT sensors. As shown in Figure 20a, the adhesive layers were simulated with the thickness of 0.1 mm for both the PZT and the interface. The whole FE model was meshed by 4155 elastic blocks.

The material properties of the interface, the connection splice, and the adhesive layer are shown in Table 3, and the piezoelectric properties of the PZT sensor (PZT-5A type) can be found in an existing publication [29]. The contact stiffness was set as *k_x_* = *k_y_* = 2.5 × 10^11^ N/m^2^/m and *k_z_* = 5.0 × 10^11^ N/m^2^/m for the healthy state. The effect of damping is represented by the damping loss factor *η*, shown in Table 3. For acquiring the EM impedance from the PZT interface, a harmonic excitation voltage with an amplitude of 1 V was applied to the top surface of the PZT patch, while the bottom was set as the ground electrode. It is noted that the value of contact stiffness was assumed to be unchanged during the PZT excitation. This is because the simulation aimed to only quantify the variation of impedance responses induced by the damage.

#### 4.1.2. Simulation Cases

**Figure 21:**
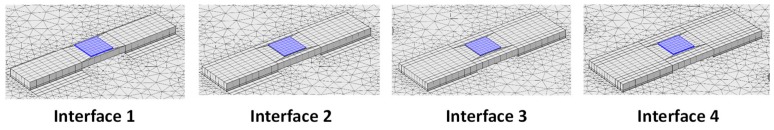
Case 1: FE simulation of Interfaces 1–4.

Two simulation cases (i.e., Case 1 and Case 2) following the experiment were investigated. The first simulation case (Case 1) was performed to numerically analyze the effect of the interface’s size on the sensitivity of impedance signatures. Accordingly, the four different interfaces (Interfaces 1–4) of the experiment were simulated, as shown in Figure 21. Interfaces 1–4 were modeled according to their actual sizes. The impedance signatures of the four PZT interfaces were numerically analyzed under an intact state and a damage event (i.e., the 12.5% loss of the contact stiffness). 

**Figure 22:**
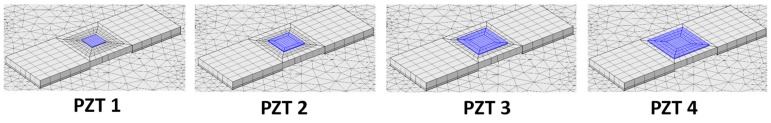
Case 2: FE simulation of PZTs 1–4.

The second simulation case (Case 2) was conducted to numerically examine the effect of the PZT’s size on the sensitivity of the impedance signatures. The four different PZT sensors (PZTs 1–4) of the experiment were simulated, as shown in Figure 22. The EM impedance signatures in 10–55 kHz of the PZTs 1–4 were analyzed under the intact state and with the 12.5% loss of contact stiffness. 

### 4.2. Numerical Analysis of Interface’s Geometric Parameters

#### 4.2.1. Numerical Impedance Signatures versus Interface Width

**Figure 23:**
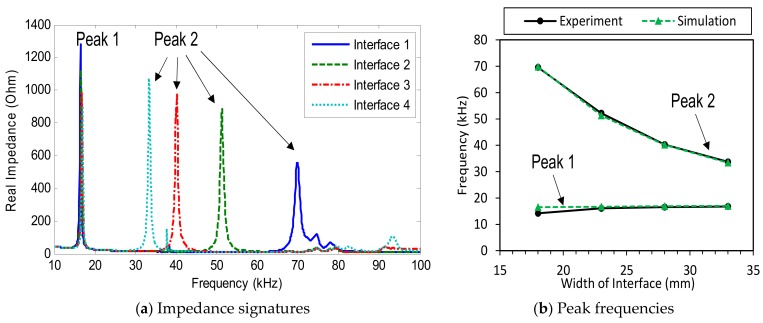
Numerical impedance signatures of FE model: Interfaces 1–4.

The numerical impedance in 10–100 kHz (901 swept points) obtained from the four interfaces under the intact case is shown in Figure 23a. Comparing Figure 23a with Figure 4a, we observed the disagreement in the impedance magnitudes between the simulation and the experimental results. This could be induced by the differences in the modal damping between the experiment model and the FE model. Despite that, the peak frequencies between the two models were in good agreement, as shown in Figure 23b. It is also noted that the peak frequencies were only slightly affected by damping values.

For each interface, two clear impedance peaks (i.e., Peak 1 and Peak 2) can be observed in Figure 23a. The first peaks (Peak 1) of the four interfaces were very close in their frequencies, while the second peaks (Peak 2) were quite different from one another. When the interface’s width was increased from 18 mm to 33 mm, the frequency of Peak 1 was slightly increased, from 16.5 kHz to 17 kHz, while that of Peak 2 was significantly reduced from about 69.8 kHz to around 33.4 kHz. This observation was consistent with the experimental result. The peak frequencies from the simulation are compared with those from the experiment, as shown in Figure 23b. As observed from the figure, the numerical peak frequencies were well-matched with the experimental ones. 

**Figure 24:**
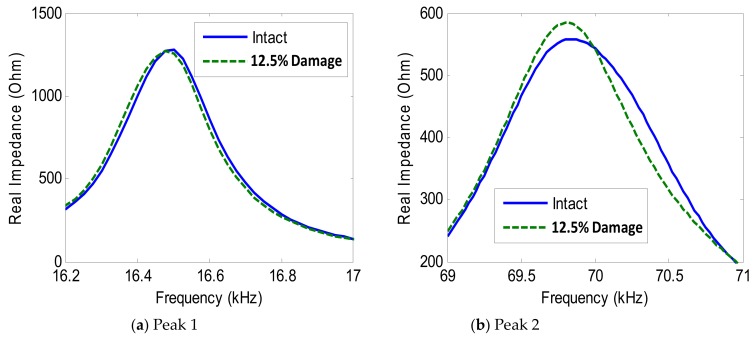
Numerical impedance signatures under damage: Interface 1.

Figure 24 shows the impedance variation of Peak 1 and Peak 2 under the damage condition (i.e., 12.5% contact stiffness loss) for Interface 1. As observed from the figure, the impedance signatures of Peak 1 and Peak 2 tended to shift left when the contact stiffness of the connection was reduced, indicating the reduction of the modal stiffness of the model.

#### 4.2.2. Damage Sensitivity of Numerical Impedance Signatures

**Figure 25:**
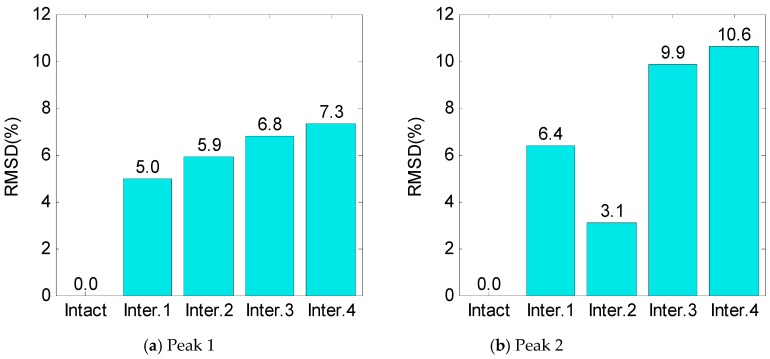
RMSD index of numerical impedance signatures under damage.

For Interfaces 1–4, the RMSD index of impedance signatures was computed as shown in Figure 25. Overall, the RMSD indices of the two impedance peaks (Peak 1 and Peak 2) increased according to the increment in the interface’s width. As compared to Peak 1, Peak 2 showed relatively higher variations in the RMSD index. This observation was well-matched with the experimental result.

**Figure 26:**
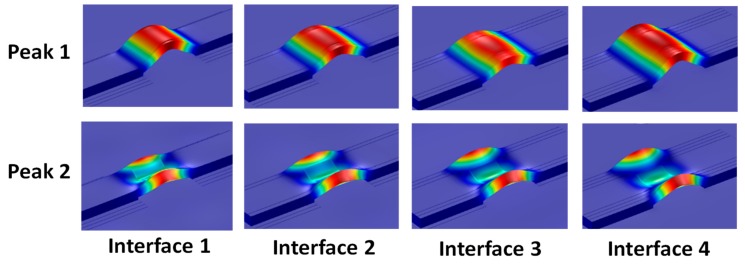
Numerical flexural motions of Peak 1 and Peak 2 of Interfaces 1–4.

Figure 26 shows the longitudinal flexural motion of Peak 1 and the lateral flexural motion of Peak 2 for Interfaces 1–4. As the interface’s width was changed from 18 mm to 33 mm, the longitudinal flexural motion of Peak 1 slightly changed, while the lateral flexural motion of Peak 2 became much more distinct. This is because the flexible section of the interface was transformed from a beam-like structure (Interface 1) to a plate-like structure (Interface 4). The more distinct motions of the impedance peaks might cause improved sensitivity of the impedance signatures.

**Figure 27:**
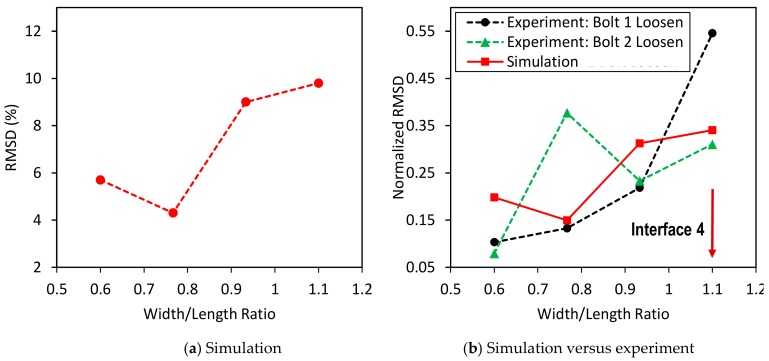
Damage sensitivity of numerical impedance signatures versus interface size.

To estimate the suitable width of the interface, the RSMD index was computed using the numerical impedance signatures in 10–100 kHz. The RMSD index was plotted versus the width/length (*B/L*) ratio of the flexible section, as shown in Figure 27a. It is observed that the damage sensitivity of impedance signatures was enhanced by increasing the width/length (*B/L*) ratio. Figure 27b compares the normalized RMSD indices of the experiment and the simulation. Similar to the experiment, the simulation also indicated that Interface 4, which has a nearly square plate-type flexural section, has the best performance for impedance monitoring. 

### 4.3. Numerical Analysis of PZT Size

#### 4.3.1. Numerical Impedance Signatures versus PZT Size

**Figure 28:**
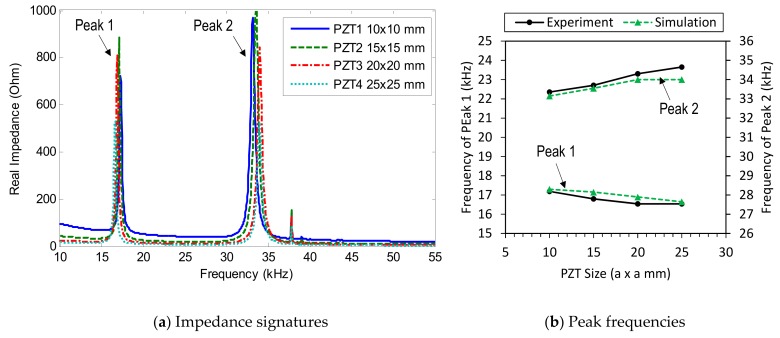
Numerical impedance signatures of the FE model: PZTs 1–4.

Figure 28a shows the numerical impedance in 10–55 kHz (901 swept points) measured for PZTs 1–4 under the intact condition. Similar to the experimental results in Figure 12a, the pattern of the numerical impedance was shifted down as the PZT size was increased. Two impedance peaks (i.e., Peak 1 and Peak 2) exist in the examined frequency range. It can be seen that the numerical impedance peaks of the four PZT sensors were very close in their frequencies, which were observed in the previous experiment.

**Figure 29:**
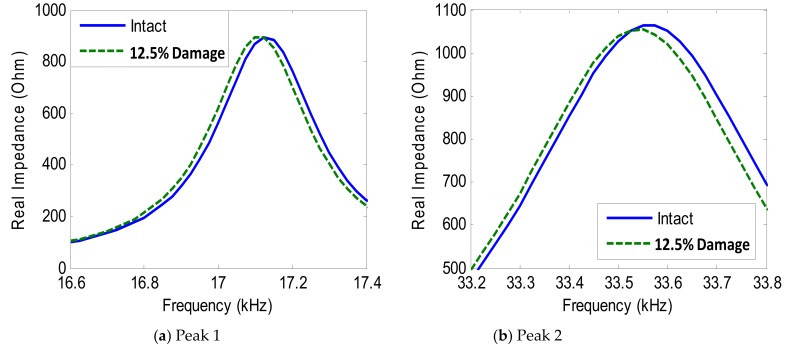
Numerical impedance signatures under damage: PZT 2.

When the PZT size was increased from 10 mm × 10 mm (PZT 1) to 25 mm × 25 mm (PZT 4), the first peak frequency (Peak 1) slightly decreased, while the second one (Peak 2) slightly increased, as observed in Figure 28b. It was also found that the peak frequencies from the simulation were well identical to those from the experiment. Figure 29 shows the variation of Peak 1 and Peak 2 obtained from PZT 2 under the damage event (i.e., 12.5% contact stiffness loss). As observed from the figure, Peak 1 and Peak 2 tended to shift left when the contact stiffness decreased.

#### 4.3.2. Damage Sensitivity of Numerical Impedance Signatures

**Figure 30:**
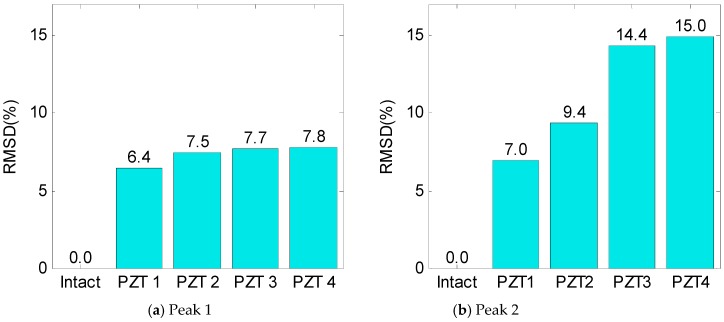
RMSD index of impedance signatures under damage: PZTs 1–4.

For PZTs 1–4, the RMSD of the impedance signatures was quantified, as shown in Figure 30. It is clear that the RMSD indices of the two impedance peaks increased according to the increment in the PZT’s size. As compared to Peak 1, Peak 2 showed relatively higher variations in the RMSD index. 

**Figure 31:**
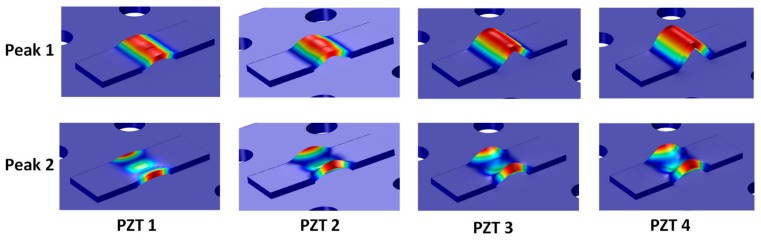
Numerical flexural motions of Peak 1 and Peak 2: PZTs 1–4.

As the PZT was increased from 10 mm × 10 mm (PZT 1) to 25 mm × 25 mm (PZT 4), the PZT driving force on the interface was also increased 6.25 times, computed based on reference [36]. This increased piezoelectric driving force would make stronger excitations to the PZT interface, resulting in the higher sensitivity of the PZT interface to the damage. As proved in Figure 31, the flexural motions of the two peaks became more distinct as the PZT size increased. 

**Figure 32:**
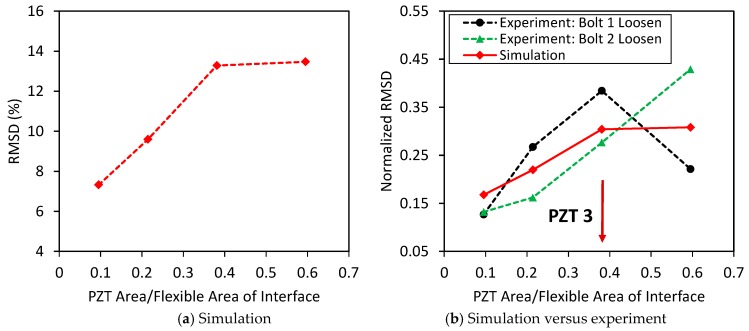
Damage sensitivity of numerical impedance signatures versus PZT size.

To estimate the suitable size of the PZT sensor, the RSMD index was computed using the numerical impedance signatures in 10–55 kHz. The damage index was plotted according to the ratio of surface areas between the PZT and the flexible section, as shown in Figure 32a. Obviously, the damage sensitivity of the impedance signatures was enhanced by increasing the PZT’s size. Figure 32b compares the normalized RMSD indices between the experiment and the simulation. Similar to the experiment, the simulation results indicated that PZT 3, which covers about 40% of the flexible section’s surface, showed the best performance for impedance monitoring. 

Conclusively, the results of the experimental model agreed well with the FE model. Therefore, the FE results verified the experimental results, and also demonstrated that the experimental model was set up with acceptable errors.

## 5. Conclusions

This study presents a set of experimental and numerical investigations to study the effect of the PZT interface’s geometric parameters on the damage sensitivity of the EM impedance. From the experimental and numerical investigations on a bolted connection, the following concluding remarks can be drawn, as follows:

(1) When the width of the interface increased, the flexible section of the interface changed from the beam-like structure to the plate-like structure. As a result, the resonant frequency of Peak 1 was slightly increased, while the resonant frequency of Peak 2 was significantly decreased. The longitudinal flexural motion of Peak 1 remained unchanged, while the lateral flexural motion of Peak 2 became more distinct, along with an increase of the interface’s width. The more distinct motions of the impedance peaks might cause improved sensitivity of the impedance signatures.
(2) When the size of the PZT was increased, the impedance pattern shifted down, and the resonant frequency of Peak 1 slightly decreased, while the resonant frequency of Peak 2 slightly increased. The PZT driving force on the interface increased, along with an increase in the PZT size, making the flexural motions of the two peaks became more distinct. This increased piezoelectric driving force would make stronger excitations to the PZT interface that could result in higher damage sensitivity of the PZT interface.
(3) To maximize its damage detection performance, the PZT interface should be designed to have the flexible section of a near-square shape, and the PZT patch covering 40% of the surface area of the flexible section.

These obtained results above can be used for other bolted structures with similar configurations. For future study, a more complicated simulation should be implemented into the FE model to assess the stiffness change during by the PZT vibration. Also, the comprehensive analysis of the bonding effect on the impedance characteristics will be investigated.

## Figures and Tables

**Table 1:** Geometric parameters of PZT interfaces

Case 1: Different Interfaces with Same PZT			Case 2: Different PZTs on the Same Interface		
Parameters	*a*	*B*	Parameters	*a*	*B*
Interface 1	15 mm	18 mm	PZT 1	10 mm	33 mm
Interface 2	15 mm	23 mm	PZT 2	15 mm	33 mm
Interface 3	15 mm	28 mm	PZT 3	20 mm	33 mm
Interface 4	15 mm	33 mm	PZT 4	25 mm	33 mm

**Table 2:** Bolt-loosening events of the bolted connection.

Loosened Bolt	Description of Bolt Torque Level (Nm)
Bolt 1	Bolt 1: 160 → 110 (−31%) → 60 (−62%) → 0 (−100%); all others: 160
Bolt 2	Bolt 2: 160 → 110 (−31%) → 60 (−62%) → 0 (−100%); all others: 160

**Table 3:** Material properties of splice plate and PZT interface [29].

Parameters	PZT Interface	Connection Splice	Adhesive Layer
Young’s modulus, *E* (GPa)	70	200	6
Poisson’s ratio, *υ*	0.33	0.3	0.38
Mass density, *ρ* (kg/m^3^)	2700	7850	1700
Damping loss factor, *η*	0.02	0.02	0.02
